# Multiplex serology demonstrate cumulative prevalence and spatial distribution of malaria in Ethiopia

**DOI:** 10.1186/s12936-019-2874-z

**Published:** 2019-07-22

**Authors:** Ashenafi Assefa, Ahmed Ali Ahmed, Wakgari Deressa, Heven Sime, Hussein Mohammed, Amha Kebede, Hiwot Solomon, Hiwot Teka, Kevin Gurrala, Brian Matei, Brian Wakeman, G. Glenn Wilson, Ipsita Sinha, Richard J. Maude, Ruth Ashton, Jackie Cook, Ya Ping Shi, Chris Drakeley, Lorenz von Seidlein, Eric Rogier, Jimee Hwang

**Affiliations:** 1 Ethiopian Public Health Institute, Addis Ababa, Ethiopia; 2 School of Public Health, Addis Ababa University, Addis Ababa, Ethiopia; 3 African Society for Laboratory Medicine, Addis Ababa, Ethiopia; 4 Ethiopian Federal Ministry of Health, Addis Ababa, Ethiopia; 5 U.S. President’s Malaria Initiative, United States Agency for International Development, Addis Ababa, Ethiopia; 6 Malaria Branch, Division of Parasitic Diseases and Malaria, Centers for Disease Control and Prevention, Atlanta, GA USA; 7 Department of Biology, University of Southern Denmark, 5230 Odense M, Denmark; 8 Mahidol Oxford Research Unit, Mahidol University, Bangkok, Thailand; 9 Centre for Tropical Medicine and Global Health, Nuffield Department of Medicine, University of Oxford, Oxford, UK; 10 Harvard TH Chan School of Public Health, Harvard University, Boston, USA; 11 Center for Applied Malaria Research and Evaluation, Tulane School of Public Health and Tropical Medicine, New Orleans, LA USA; 12 London School of Hygiene and Tropical Medicine, London, UK; 13 Malaria Branch, Division of Parasitic Diseases and Malaria, U.S. President’s Malaria Initiative, Centers for Disease Control and Prevention, Atlanta, GA USA

**Keywords:** Multiplex serology, Seroprevalence, Malaria, Ethiopia

## Abstract

**Background:**

Measures of malaria burden using microscopy and rapid diagnostic tests (RDTs) in cross-sectional household surveys may incompletely describe the burden of malaria in low-transmission settings. This study describes the pattern of malaria transmission in Ethiopia using serological antibody estimates derived from a nationwide household survey completed in 2015.

**Methods:**

Dried blood spot (DBS) samples were collected during the Ethiopian Malaria Indicator Survey in 2015 from malarious areas across Ethiopia. Samples were analysed using bead-based multiplex assays for IgG antibodies for six *Plasmodium* antigens: four human malaria species-specific merozoite surface protein-1 19kD antigens (MSP-1) and Apical Membrane Antigen-1 (AMA-1) for *Plasmodium falciparum* and *Plasmodium vivax*. Seroprevalence was estimated by age, elevation and region. The seroconversion rate was estimated using a reversible catalytic model fitted with maximum likelihood methods.

**Results:**

Of the 10,278 DBS samples available, 93.6% (9622/10,278) had valid serological results. The mean age of participants was 15.8 years and 53.3% were female. National seroprevalence for antibodies to *P. falciparum* was 32.1% (95% confidence interval (CI) 29.8–34.4) and 25.0% (95% CI 22.7–27.3) to *P. vivax*. Estimated seroprevalences for *Plasmodium malariae* and *Plasmodium ovale* were 8.6% (95% CI 7.6–9.7) and 3.1% (95% CI 2.5–3.8), respectively. For *P. falciparum* seroprevalence estimates were significantly higher at lower elevations (< 2000 m) compared to higher (2000–2500 m) (aOR 4.4; p < 0.01). Among regions, *P. falciparum* seroprevalence ranged from 11.0% (95% CI 8.8–13.7) in Somali to 65.0% (95% CI 58.0–71.4) in Gambela Region and for *P. vivax* from 4.0% (95% CI 2.6–6.2) in Somali to 36.7% (95% CI 30.0–44.1) in Amhara Region. Models fitted to measure seroconversion rates showed variation nationally and by elevation, region, antigen type, and within species.

**Conclusion:**

Using multiplex serology assays, this study explored the cumulative malaria burden and regional dynamics of the four human malarias in Ethiopia. High malaria burden was observed in the northwest compared to the east. High transmission in the Gambela and Benishangul-Gumuz Regions and the neglected presence of *P. malariae* and *P. ovale* may require programmatic attention. The use of a multiplex assay for antibody detection in low transmission settings has the potential to act as a more sensitive biomarker.

**Electronic supplementary material:**

The online version of this article (10.1186/s12936-019-2874-z) contains supplementary material, which is available to authorized users.

## Background

Malaria transmission in Ethiopia is non-stable, heterogeneous and seasonal, with peak periods following the main rains from June to September and short rains from March to April [1]. Historically, malaria epidemics occurred at 5 to 8-year intervals. The last malaria epidemic in 2004/2005 caused two million clinical cases and claimed approximately 3000 lives [2, 3]. Although malaria transmission has been declining over the past 15 years [4, 5], 60% of the Ethiopian landmass is suitable for malaria transmission and communities remain vulnerable for malaria transmission. The National Malaria Control Programme of Ethiopia considers areas below 2000 m elevation as malaria endemic and eligible for malaria control activities [6]. *Plasmodium falciparum* and *Plasmodium vivax* are the major documented malaria parasite species, with approximately 1.2 million *P. falciparum* cases and 678,000 *P. vivax* cases reported in 2015 [7]. The proportion of *P. vivax* cases has been increasing whereas a decline in *P. falciparum* incidence was observed [8].

With the decline in malaria cases, measuring malaria transmission intensity and dynamics is becoming increasingly challenging. Transmission estimates using standard malaria diagnostic tests, such as microscopy or rapid diagnostic tests (RDT), entomological inoculation rate, and splenomegaly prevalence fail to detect changes in the malaria burden in very low transmission settings [9]. The last three Ethiopian Malaria Indicator Surveys (EMIS) reported very low prevalence, using microscopy and RDTs: 0.9% by microscopy in 2007, 1.3% by microscopy and 4.5% by RDTs in 2011, and 0.5% by microscopy and 1.2% by RDT in 2015 [10–12]. With decreasing parasite prevalence as well as density of individual infections, the sensitivity of conventional diagnostic tools has been declining [13, 14]. Serological and molecular epidemiological studies may be more useful in such scenarios, and are being increasingly used for measuring malaria burden in low transmission settings [15–17]. Robust seroprevalence estimates of *Plasmodium* infections could be critical for monitoring and evaluation of ongoing malaria control and elimination activities in Ethiopia.

In this study, multiplex serological methods were used to estimate malaria exposure, transmission patterns, and spatial and regional distribution of malaria in Ethiopia, using samples collected during the 2015 EMIS.

## Methods

### Study area

The study was conducted as part of the National Ethiopian Malaria Indicator Survey in 2015 (EMIS-2015). Ethiopia has successfully implemented three national EMISs in 2007, 2011 and 2015 [10–12]. EMIS-2015 was embarked during the peak of malaria transmission season, between September and December 2015. The sampling design provided national and sub-national estimates of major malaria intervention indicators, including malaria prevalence for malarious areas of the country, as well as estimates for areas below 2000 m elevation, areas between 2000 and 2500 m, and for 10 administrative regions.

### Study design and sample collection

EMIS-2015 was a cross-sectional survey that used a two-stage cluster sampling approach. A total of 555 enumeration areas (EAs) were selected proportional to the population size of the regions, as estimated by the Ethiopian Central Statistics Agency. In each EA, 25 households were randomly selected following onsite EA household mapping. A total of 53,335 individuals were surveyed in 13,875 selected households. Demographic, socio-economic, use of malaria prevention, and malariometric data were collected in the selected households from each EA. Children under 5 years of age in each selected household and persons of all ages in every fourth household were eligible for biological sample testing. Whole blood from a finger prick from consenting individuals was collected for a malaria RDT (histidine‐rich protein 2 (HRP2) and *Plasmodium* lactate dehydrogenase (pLDH) antigen CareStart^®^, AccessBio, US), a malaria blood slide (thick and thin blood film), haemoglobin (Hemocue Hb 201+ , Hemocue AB, Ängelholm, Sweden) measurement, and dried blood spot (DBS) samples. Whatman 903 Protein Saver (GE Healthcare, Pittsburgh, PA, USA) filter paper was used for DBS collection. Filter papers were air dried, individually packed in a plastic bag together with a desiccant and stored at − 20 °C at the Ethiopian Public Health Institute (EPHI) before they were shipped for further laboratory-based testing at the US Centers for Disease Control and Prevention (CDC), Atlanta, GA, USA.

### Ethical consideration

The EMIS-2015 protocol received ethical clearance from the National Research Ethics Review Committee of Ethiopia and PATH. The survey protocol underwent human subjects review at CDC and was considered to be a non-research programme evaluation activity. Additional ethical clearance for the present serology study was obtained from the Institutional Review Board of the College of Health Sciences of the Addis Ababa, University (AAUMF 03-008).

### Blood spot elution, bead coupling and antigens used in the study

Laboratory analysis was conducted as previously described [15]. Briefly, a 6-mm punch was used from each DBS sample and blood eluted to a 1:20 concentration in blocking buffer (Buffer B: Phosphate Buffered Saline (PBS) containing 0.5% Bovine Serum Albumin (BSA), 0.05% Tween 20, 0.02% sodium azide, 0.5% polyvinyl alcohol, 0.8% polyvinylpyrrolidone and 0.5% w/v *Escherichia coli* extract) overnight.

Six *Plasmodium* antigens were used for this study: two *P. falciparum* antigens (Merozoite Surface Protein-1 19kD (MSP-1) and Apical Merozoite Antigen-1 (AMA-1), two *P. vivax* antigens (PvMSP-1 and PvAMA-1), one *P. malariae* (PmMSP-1) and one *P. ovale* (PoMSP-1). The four *Plasmodium* MSP-1 19kD antigens were produced as recombinant proteins and purified as described previously [18]. The external domain of *P. falciparum* AMA-1 antigen was produced at the Walter Reed Army Institute of Research (WRAIR) under previously published conditions [19]. The *P. vivax* AMA-1 antigen was produced at the London School of Hygiene and Tropical Medicine (LSHTM) under previously published conditions [20]. The *Schistosoma japonicum* glutathione-*S*-transferase antigen (GST) was produced recombinantly and served as a generic protein to assess immunoglobulin G (IgG) non-specific binding.

All antigens were coupled to magnetic beads (Luminex Corp, Austin, TX, USA) as per prior studies [18, 21]. Briefly, beads were pulse vortexed, transferred to a microcentrifuge tube and centrifuged for 1.5 min at 13,000*g*. Supernatant was removed, and the beads were washed with a 0.1 M and pH 6.2 sodium phosphate (NaP) solution. Beads were activated by suspending in NaP with 5 mg/mL of EDC (1-ethyl-3-[3-dimethylaminutesopropyl] carbodiimide hydrochloride) and 5 mg/mL sulfo-NHS (sulfo N-hydroxylsulfosuccinimide) and incubating with rotation for 20 min at room temperature (RT), while protected from light. After a wash with coupling buffer (50 mM 2-(4-morpholino)-ethane sulfonic acid, 0.85% NaCl at pH 5.0), antigens were coupled to beads in the presence of a coupling buffer for 2 h at a concentration of 20 ug/mL for all antigens, except for PvAMA1 and GST at 15 ug/mL. Beads were washed once with PBS and suspended in PBS with 1% bovine serum albumin (BSA) with incubation for 30 min at RT by rotation. Beads were then resuspended in a storage buffer (PBS, 1% BSA, 0.02% sodium azide and 0.05% Tween-20) and stored at 4 °C. Coupled beads were run with a panel of known malaria seronegatives to assure minimal non-specific MFI signal would be given by beads used in this study.

The DBS elution was assayed for IgG antibodies using bead-based multiplex technology, and all wash steps were performed with a handheld magnet. In 5 mL of reagent buffer (Buffer A: PBS, 0.5% BSA, 0.05% Tween-20, 0.02% NaN_3_), a bead mix was prepared with all coupled bead regions included (approximately 625 beads/antigen per well), and 50 uL bead mix was pipetted into each well of a BioPlex Pro plate (BioRad, Hercules, CA, USA). Beads were washed twice with 100 µL PBS with Tween (PBST), and 50 µL of the reagent mix [in 5 mL Buffer A: 1:500 anti-human IgG (Southen Biotech), 1:625 anti-human IgG_4_ (Southern Biotech), 1:200 streptavidin-PE (Invitrogen)] was added to each well. Negative control samples and a dilution curve of hyperimmune serum were added to each assay plate to monitor any change in control values over the course of the study. Samples of blood eluted from DBS were added to predefined wells (already containing beads and reagent mix) at 1:50 dilution. Plates were incubated overnight with gentle shaking at RT and protected from light. The next morning (approximately 16 h total incubation time), plates were washed three times, and beads were re-suspended with 100 µL PBS and read on a MAGPIX machine (Luminex Corp, Austin, TX, USA). Mean fluorescence intensity (MFI) signal was generated for a minimum of 50 beads/region, and the background (bg) MFI from wells incubated with Buffer B was subtracted from each sample to give a final value of MFI-bg. Minimal variation was seen in the assay signal for the background or positive hyperimmune serum curve values, so no plates were re-run for the study.

### Statistical analysis

Data analysis was done using Stata 13 software (College Station, USA). Samples with GST MFI-bg reads above 1000 MFI value (non-specific binding) were excluded from the analysis (n = 678). To dichotomize seropositivity, log_10_-transformed MFI-bg values were fitted to a two-component Finite Mixture Model (FMM) by the FMM procedure with normal distribution and maximum likelihood estimation outputs. A seropositivity cutoff value was determined by the mean MFI-bg value of the first (assumed seronegative) component plus three standard deviations [18]. Overall, *P. falciparum* and *P. vivax* seropositivity were defined as an individual being positive for either or both of the MSP-1 and AMA-1 antigens for each species. Seropositivity estimates for *P. malariae* and *P. ovale* are presented using the seropositivity cut-off method outlined above. However, to minimize cross-reactivity between the species-specific MSP-1 antigens, the current study report an additional conservative two-stage approach that increase specificity, but decrease sensitivity of the specific antibodies to the targeted antigens. For this conservative approach for *P. malariae* and *P. ovale*, individual readings for PmMSP-1 or PoMSP-1 response first had to be above the MFI-bg cutoff as described above. Additionally, the PmMSP-1 and/or PoMSP-1 MFI-bg signal for that sample also needed to be above the PfMSP-1 MFI-bg signal for the same sample (ratio to PfMSP-1 greater than 1.0) to be considered PmMSP-1 and/or PoMSP-1 positive.

A reversible catalytic model was fitted to the dichotomized data using maximum likelihood methods to generate a seroconversion rate (SCR or λ) and a seroreversion rate (ρ). The model was used to generate age seroprevalence curves, from which a seroconversion rate (SCR) representing the force of infection for the community was calculated. Evidence for two forces of infection was investigated, if visual inspection of SCR curves indicated such a comparison and was guided by a profile likelihood plots to determine the most likely time (year) of change in transmission [16, 22]. Multiple logistic regression models were employed to determine odds ratios (OR); 95% confidence intervals (CI) for gender, age and elevations. Similar regression models were employed for *P. falciparum* and *P. vivax* seropositivity. Adjustments were made by region, elevation and age group.

Empirical bayesian kriging in ArcGIS version 10.5 (ESRI, CA, USA) was used to predict the spatial distribution of seroprevalence as a continuous surface of probability of being above a cut-off. This is an established method that uses a statistical model to predict and interpolate the spatial distribution of a variable from available data [23]. Maps were developed separately for MSP-1 and AMA-1 antigens for both *P. falciparum* and *P. vivax* for all individuals and for children under 5 years of age. Seroprevalence by *woreda*/district was compared with the 2015 annual parasite incidence (API) data. QGIS version 2.1.8 and ArcGIS version 10.5 (ESRI, Redlands, CA) were used to produce maps.

Sampling weights calculated during MIS 2015 were used to ensure the representativeness of the samples tested to the study population.

## Results

### Study population

**Fig. 1:**
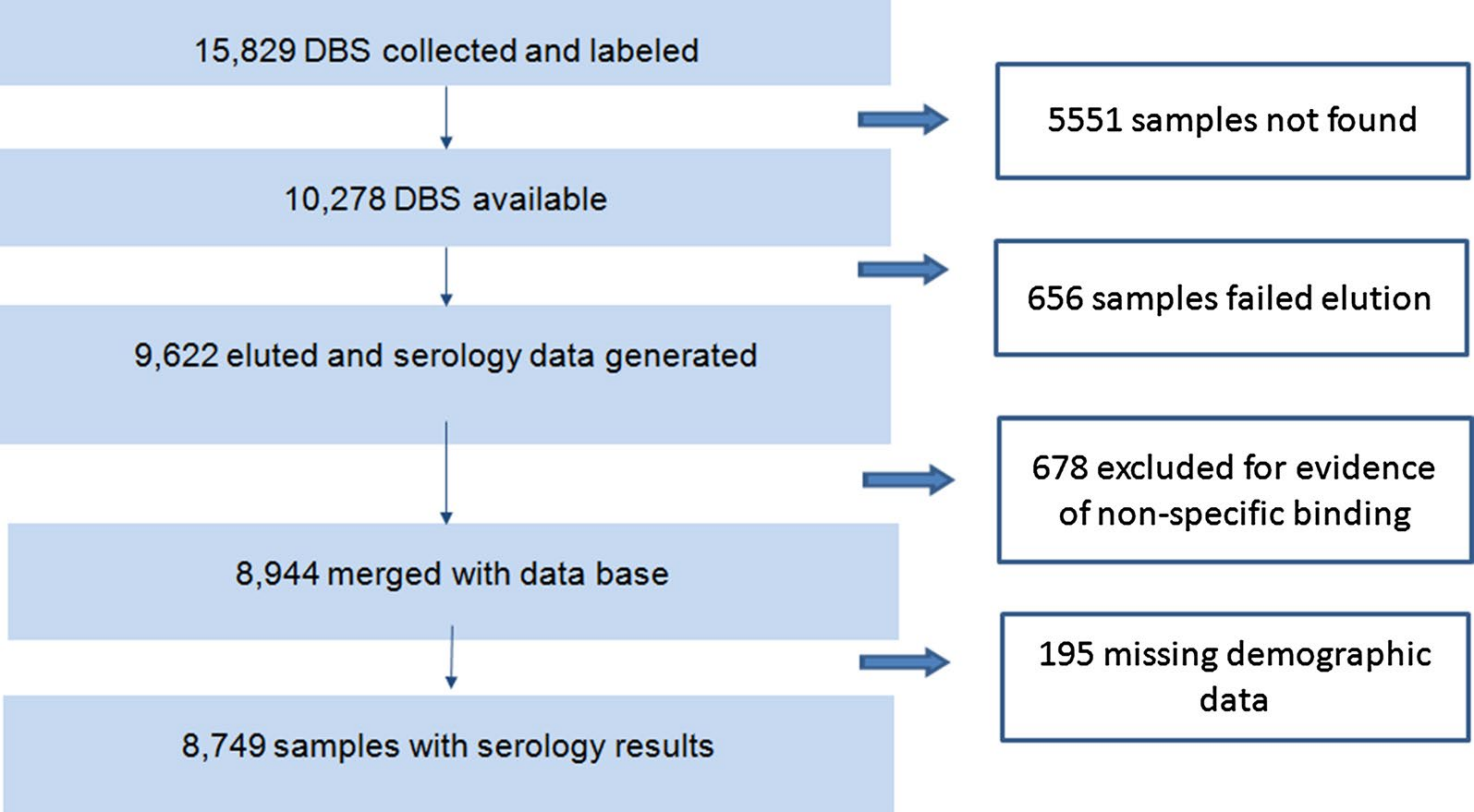
Flow chart showing sample selection and analysis procedure

**Fig. 2:**
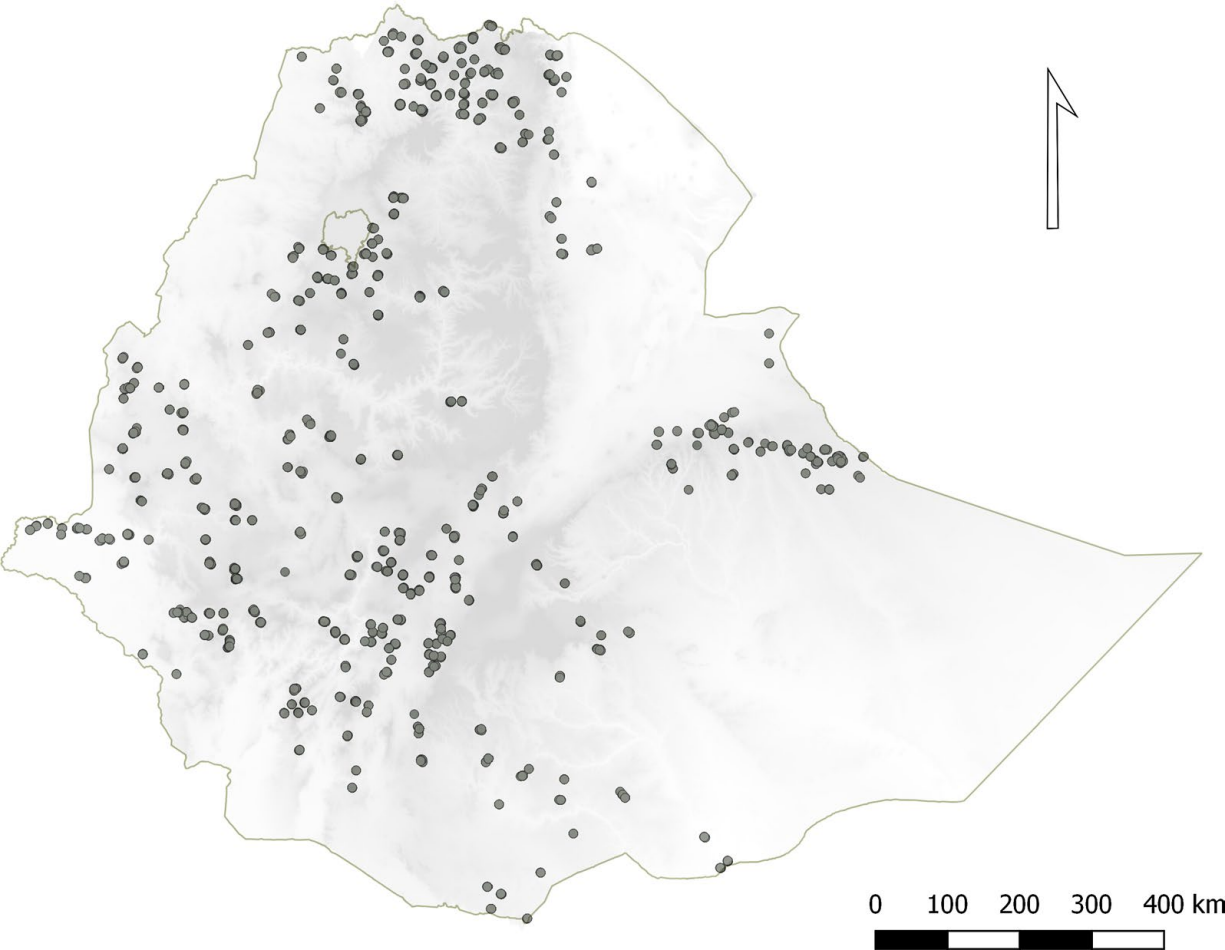
Distribution of dried blood spot (DBS) samples collected from selected malaria endemic areas of Ethiopia as part of the national, cross-sectional, household Malaria Indicator Survey conducted in 2015 (EMIS-2015). Each point depicts a cluster of sampled households in an EA. Shading indicates elevation which ranges from − 131 m below sea level (lightest) to 4092 m (darkest)

Of the expected 15,829 DBS samples, 10,278 individual DBSs were available for the current study, serological results were generated for 93.6% (9622/10,278) of these (Fig. 1). A total of 8944 results were merged with the EMIS-2015 data base, of which 52.3% (95% CI (50.8–53.7)) of the survey participants were female (Table 1). Figure 2 shows the spatial distribution of EAs providing serology data. The overall proportion of samples collected from children under 5 years was 44.8% (95% CI 43.0–46.5); although this proportion was above 60% in the Afar and Somali Regions (Table 1). The number of individuals with serology data within an EA ranged from 1 to 55, with a mean of 24 individuals per EA. Overall mean age was 15.8 years (95% CI 15.2–16.4) (range 1–107). Of the samples collected, 75.0% (95% CI 67.5–81.2) were from areas with elevation less than 2000 m.

**Table 1:** Characteristics of study participants by administrative region, Ethiopian Malaria Indicator Survey-2015 (EMIS-2015)

	Tigray	Afar	Amhara	Oromia	Somali	Benishangul-Gumuz	Southern Nations and Nationalities Peoples’	Gambela	Harari	Diredawa	National
Gender % (95% CI)											n = 8743
Male	46.5 (44.1–48.9)	47.6 (42.9–52.3)	50.9 (47.4–54.3)	48.2 (45.6–50.9)	45.1 (40.8–49.4)	45.3 (40.8–49.9)	46.4 (44.3–48.5)	41.4 (37.4–45.5)	47.4 (3.0–65.4)	40.5 (33.1–48.4)	47.7 (46.3–49.2)
Female	53.3 (51.1–55.9)	52.4 (47.7–57.1)	49.1 (45.7–52.6)	51.8 (49.1–54.4)	54.9 (50.6–59.2)	54.7 (50.1–59.2)	53.6 (51.5–55.7)	58.6 (54.5–62.6)	52.6 (34.6–70.0)	59.5 (51.6–66.9)	52.3 (50.8–53.7)
Age group (years)% (95% CI)											
0–5	43.8 (39.6–48.0)	66.6 (63.7–69.3)	40.8 (37.1–44.7)	44.6 (41.5–47.9)	61.7 (58.8–64.6)	49.5 (41.5–57.5)	45.4 (43.2–47.7)	54.8 (50.8–58.8)	25.5 (12.9–44.2)	46.0 (35.3–57.1)	44.8 (43.0–46.5)
5–15	17.7 (15.2–20.4)	12.8 (9.2–17.5)	20.5 (18.0–23.3)	18.8 (15.8–22.3)	14.6 (11.7–18.1)	16.0 (12.9–19.6)	20.8 (18.9–22.9)	18.9 (16.6–21.5)	3.5 (1.0–11.6)	10.2 (6.6–15.4)	19.4 (17.8–21.1)
15–25	13.3 (11.3–15.6)	6.1 (3.9–9.4)	12.6 (9.5–16.6)	11.6 (10.0–13.4)	5.2 (3.6–7.5)	11.5 (7.8–16.6)	11.4 (9.7–13.4)	10.7 (8.3–13.8)	22.1 (17.4–27.5)	14.0 (10.9–17.9)	11.7 (10.7–12.8)
25–50	17.1 (14.9–19.4)	14.1 (12.1–16.4)	19.2 (16.2–22.6)	19.7 (17.5–22.1)	14.6 (13.0–16.4)	14.5 (10.7–19.3)	17.1 (15.6–18.6)	12.3 (1.1–15.0)	36.8 (29.3–45.0)	21.7 (15.9–28.9)	18.5 (17.3–19.8)
50+	8.2 (6.6–10.1)	0.5 (0.1–1.5)	6.9 (5.1–9.1)	5.2 (3.9–7.0)	3.8 (2.7–5.3)	8.6 (5.3–13.5)	5.2 (4.1–6.6)	3.2 (2.3–4.5)	12.1 (7.4–19.2)	8.1 (4.5–14.2)	5.7 (4.9–6.6)
Elevation (m) % (95% CI)											n = 8740*
< 1000	5.6 (1.6–17.4)	38.0 (13.7–70.4)	0 (0.0–0.0)	0 (0.0–0.0)	28.6 (12.6–52.7)	8.2 (1.1–52.7)	0 (0.0–0.0)	88.4 (72.1–95.8	0 (0.0–0.0)	0.9 (0.1–6.7)	1.9 (1.2–2.8)
1000–2000	64.3 (46.8–78.6)	62.0 (29.6–86.3)	53.4 (33.2–72.5)	77.4 (47.3–87.4)	71.4 (47.3–87.4)	91.8 (57.4–98.9)	79.0 (67.3–87.3)	11.6 (4.2–27.9)	100 (100.0–100.0)	99.1 (93.9–99.9)	73.1 (65.6–79.4)
> 2000	30.2 (16.5–48.5)	0 (0.0–0.0)	46.6 (27.5–66.8)	22.6 (13.1–36.1)	0 (0.0–0.0)	0 (0.0–0.0)	21 (12.7–32.7)	0 (0.0–0.0)	0 (0.0–0.0)	0 (0.0–0.0)	25.0 (18.8–32.5)
Bed net use (last night) % (95% CI)											n = 5168**
Yes	57.6 (50.3–64.5)	80.5 (65.7–89.9)	59.1 (48.1–69.4)	69.6 (59.6–78.1)	75.2 (61.2–85.4)	66.1 (54.3–76.2)	55.7 (49.4–61.8)	84.7 (76.0–90.6)	33.5 (12.8–63.3)	25 (14.3–41.6)	62.1 (57.5–66.6)
No	42.4 (35.5–49.7)	19.5 (10.1–34.3)	40.9 (30.6–51.9)	30.4 (21.9–40.4)	24.8 (14.6–38.8)	33.9 (23.8–45.7)	44.3 (38.2–50.6)	15.3 (9.4–24.0)	66.5 (36.7–87.2)	74.4 (58.4–85.7)	37.9 (33.4–42.5)
RDT results % (95% CI)											
Pf	0.8 (0.4–1.6)	0 (0.0–0.0)	0.7 (0.2–1.8)	0.2 (0.1–0.6)	0 (0.0–0.0)	6.2 (2.2–16.3)	0.2 (0.1–0.5)	16.8 (11.8–23.2)	0 (0.0–0.0)	0 (0.0–0.0)	0.5 (0.3–0.7)
Pan	0.7 (0.3–1.3)	0.2 (0.0–1.3)	0 (0.0–0.0)	0.1 (0.0–0.4)	0.1 (0.0–0.8)	0 (0.0–0.0)	0.4 (0.1–1.2)	0.1 (0.0–0.6)	0 (0.0–0.0)	0 (0.0–0.0)	0.2 (0.1–0.4)
Pf/Pan	0.1 (0.0–0.6)	0 (0.0–0.0)	0.5 (0.1–1.8)	0 (0.0–0.0)	0 (0.0–0.0)	0.4 (0.0–2.6)	0.2 (0.0–0.6)	1.7 (0.9–3.0)	0 (0.0–0.0)	0 (0.0–0.0)	0.1 (0.1–0.3)
Negative	98.4 (97.4–99.0)	99.8 (98.7–100.0)	98.9 (96.7–99.6)	99.7 (99.3–99.8)	99.9 (99.2–100.0)	93.5 (82.3–97.8)	99.3 (98.3–99.7)	81.5 (74.5–86.9)	100 (100.0–100.0)	100 100.0–100.0)	99.2 (98.8–99.4)

n, number of observations; RDT, rapid diagnostic test; *3 people did not have GPS coordinates; **3613 individuals were without bed net use data; ***RDT test was not done for 4 individuals; 4 RDT tests were invalid and 3 individuals refused RDT testing

A total of 0.8% (95% CI 0.6–1.2) of the participants were positive by RDT; comprising 0.5% (95% CI 0.3–0.7) for *P. falciparum* (HRP2 antigen positive), 0.2% (95% CI 0.1–0.4) for either *P. vivax, P. malariae or P. ovale* (Pv/Pm/Po) (pan-LDH antigen positive), and 0.1% (95% CI 0.1–0.3) considered as a mixed infection (HRP2 and pan-LDH positive). More than 90% of the positive *P. falciparum* RDT results were from the Gambela and Benishangual-Gumuz Regions. 62.1% (95% CI 57.5–66.6) of respondents said they used a bed net during the previous night (Table 1).

### Seroprevalence

Nationally, 32.1% (95% CI 29.8–34.4) of the participants were seropositive for any IgG against the two *P. falciparum* antigens, and 25.0% (95% CI 22.7–27.34) for *P. vivax*. Only 6.6% (95% CI 5.5–8.0) of participants were seropositive for both *P. falciparum* and *P. vivax*, while 41.1% (95% CI 38.5–43.7) of individuals were positive either for *P. falciparum* or *P. vivax*. Eight point six percent (95% CI 7.6–9.7) and 3.1% (95% CI 2.5–3.8) were seropositive for *P. malariae* and *P. ovale*, respectively (Table 2). By antigen, 23.8% (95% CI 21.4–26.3) were positive against *P. falciparum* MSP-1 and 24.5% (95% CI 22.4–26.7) AMA-1, while for *P. vivax*, 21.2% (95% CI 19.2–23.4) were positive against MSP-1 and 14.4% (95% CI 12.7–16.2) AMA-1.

**Table 2:** Proportion of individuals (all ages and under 5 years) found to be malaria seropositive by species and by antigen in malaria endemic areas of Ethiopia, 2015

*Plasmodium* species	Antigens	% seropositive (95% CI) (all ages)	% seropositive (95% CI) (under 5 years)
*P. falciparum*	MSP-1	23.8 (21.4–26.3)	12.3 (10.6–14.2)
	AMA-1	24.5 (22.4–26.7)	11.3 (9.8–13.0)
	Pf positive (MSP-1 and/or AMA-1)	32.1 (29.8–34.4)	18.0 (16.1–20.1)
*P. vivax*	MSP-1	21.2 (19.2–23.4)	12.3 (10.5–14.4)
	AMA-1	14.4 (12.7–16.2)	6.7 (5.5–8.0)
	Pv positive (MSP-1 and/or AMA-1)	25.0 (22.7–27.3)	14.8 (12.7–17.2)
*P. malaria*e^a^	MSP-1	8.6 (7.6–9.7)	6.1 (5.0–7.3)
*P. ovale* ^a^	MSP-1	3.1 (2.5–3.8)	2.0 (1.4–2.7)
Any *Plasmodium*	Pf (MSP-1/AMA-1) or Pv (MSP-1/AMA-1) or Pm MSP-1 or Po MSP-1	44.5 (41.9–47.2)	30.2 (27.3–33.2)
Either *P. falciparum* or *P. vivax*	Pf (MSP-1/AMA-1) or Pv (MSP-1/AMA-1)	41.1 (38.5–43.7)	26.7 (24.0–29.5)
Both *P. falciparum* and *P. vivax*	Pf (MSP-1/AMA-1) and Pv (MSP-1/AMA-1)	6.6 (5.5–8.0)	1.7 (1.2–2.4)

Samples positive for Merozoite Surface Protein-1 (MSP-1) and/or Apical Membrane Antigen-1 (AMA-1) for the respective species were considered positive for that species

Pf, *Plasmodium falciparum*; Pm, *Plasmodium malariae*; Po, *Plasmodium ovale*; Pv, *Plasmodium vivax*

^a^Normal approach (mean seronegative +3sd) to defining cut-off level used for *P. malariae* and *P. ovale*

For children under 5 years of age, 18.0% (95% CI 16.1–20.1) and 14.8% (95% CI 12.7–17.2) were seropositive for *P. falciparum* and *P. vivax*, respectively. Similarly, 33.3% (95% CI 30.4–36.2) of male children were seropositive for *P. falciparum* compared to 30.9% (95% CI 28.3–33.6) of the females [aOR: 1.3 (95% CI 1.1–1.4), p < 0.05] (Additional file 1). A similar trend was observed for *P. vivax*, where 26.4% (95% CI 23.4–29.7) of males compared to 23.6% (95% CI 21.1–26.3) of females were seropositive [aOR: 1.3 (95% CI 1.1–1.5), p > 0.05].

### Seroprevalence by age

The DBS sample collection was purposely biased towards children under 5 years of age, with 44.8% (95% CI 43.0–46.5) of the DBS samples having been collected from this age group. Seropositivity increased with age group. This pattern was more pronounced for *P. falciparum* than *P. vivax*, although the difference was significant in both *Plasmodium* species (p < 0.05).

### Conservative estimate for *Plasmodium malariae* and *Plasmodium ovale*

Table 3 shows of all samples tested, 5.1% (95% CI 4.4–6.0) were seropositive for *P. malariae* and 1.5% (95% CI 1.1–2.0) for *P. ovale* using the conservative approach to define seropositivity. Both species were observed in all the regions, albeit with a variable distribution. The proportion of seropositives was slightly lower for both species, accordingly to the conservative estimation method. A similar trend of seropositivity was observed for children under 5 years of age (Table 3).

**Table 3:** Proportion seropositive for less common malarias [*P. malariae* Merozoite Surface Protein-1 (PmMSP-1) and *P. ovale* Merozoite Surface Protein-1 (PoMSP-1)], using the conservative approach to defining seropositive cut-off values, by region and age group, in malaria endemic areas of Ethiopia, 2015

Region	All ages		Under 5 years	
	PmMSP-1	PoMSP-1	PmMSP-1	PoMSP-1
Tigray	1.1 (0.6–2.3)	0.6 (0.3–1.1)	1.1 (0.4–2.9)	0.3 (0.1–1.2)
Afar	1.3 (0.6–3.0)	0.6 (0.1–2.7)	1.8 (0.7–4.2)	0.5 (0.1–4.2)
Amhara	5.0 (3.2–7.6)	2.4 (1.8–3.3)	2.7 (1.1–6.2)	1.0 (0.3–3.1)
Oromia	6.3 (5.0–7.8)	2.0 (1.3–3.0)	5.8 (4.4–7.8)	1.8 (1.0–3.3)
Somali	1.3 (0.6–2.8)	0.1 (0.0–0.8)	1.1 (0.4–2.9)	0
Benishangul-Gumuz	2.1 (1.0–4.4)	0.7 (0.2–2.5)	1.3 (0.4–4.7)	1.5 (0.4–4.9)
Southern Nation and Nationalities People’s	4.9 (3.7–6.3)	0.6 (0.4–1.2)	4.7 (3.1–7.1)	0.7 (0.3–1.8)
Gambela	0.2 (0.1–1.1)	0.1 (0.0–0.5)	0.3 (0.1–1.2)	0
Harari	4.5 (0.6–26.4)	4.0 (0.3–33.6)	0	0
Dire Dawa	2.4 (0.7–8.0)	0.8 (0.1–5.3)	1.7 (0.3–9.9)	0
Total (%)	5.1 (4.4–6.0)	1.5 (1.1–2.0)	4.6 (3.6–5.7)	1.2 (0.8–1.9)

Pm, *Plasmodium malariae;* Po, *Plasmodium ovale*

### Seroprevalence by region

**Fig. 3:**
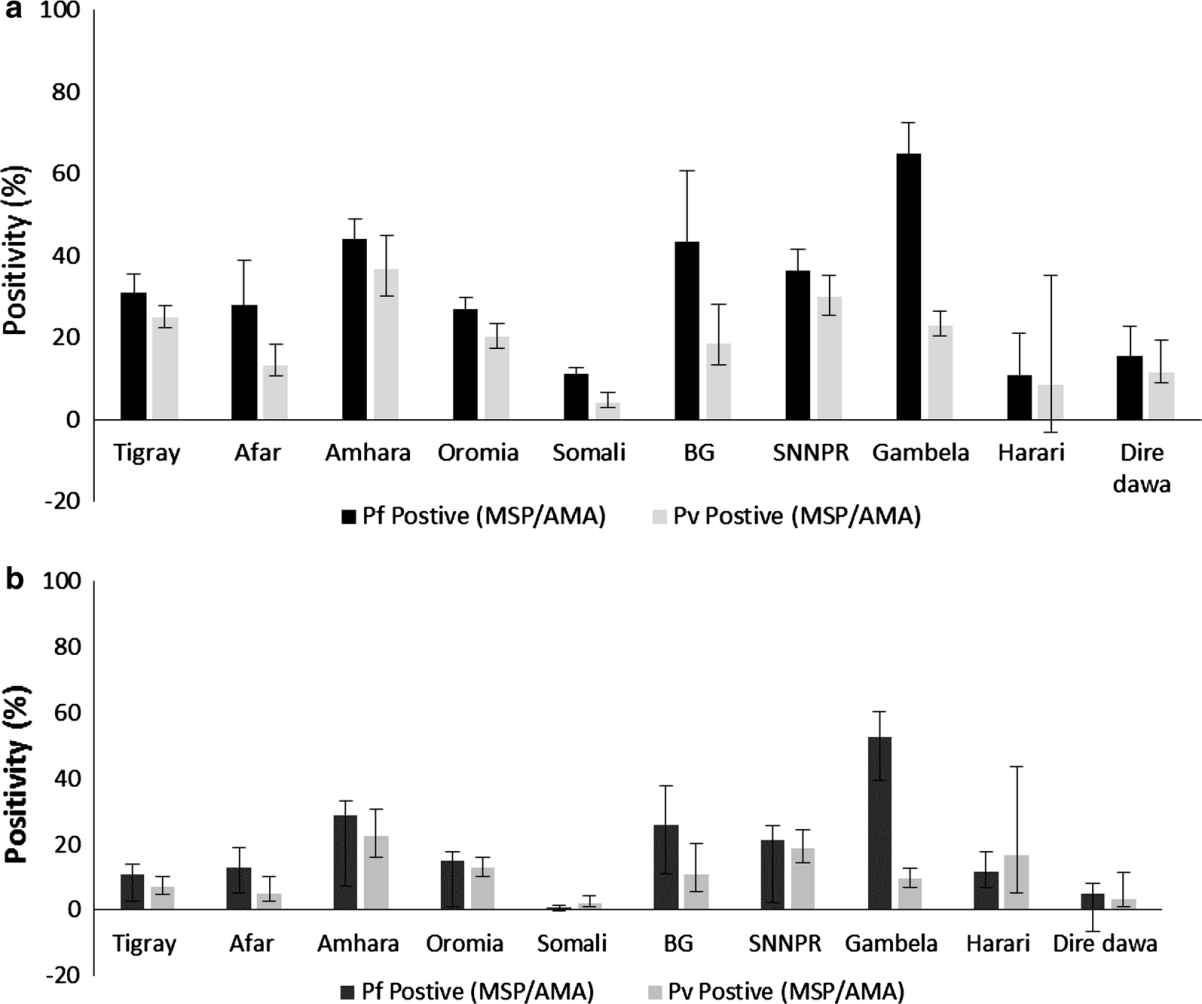
Mean seropositivity by region for *Plasmodium falciparum* and *Plasmodium vivax*, 2015. An individual was considered positive if they had a positive antibody response either for Merozoite Surface Protein-1 (MSP-1) or Apical Membrane Antigen-1 (AMA-1) antigens for each species. **a** All ages; **b** children under 5 years of age. Error bars indicate the 95% confidence interval (BG: Benishangul-Gumuz and SNNPR: Southern Nations and Nationalities People’s Region)

The proportion of individuals seropositive was variable by region, with *P. falciparum* seropositivity higher than *P. vivax* seropositivity in all the regions (Fig. 3). The proportion seropositive for *P. falciparum* by region ranged from 11.0% (95% CI: 8.8–13.7) in Somali to 65.0% (95% CI: 58.0–71.4) in Gambela Region and for P. vivax from 4.0% (95% CI: 2.6–6.2) in Somali to 36.7% (95% CI: 30.0–44.1) in Amhara Region.

The API data from 2014 (API 2014) obtained from the Federal Ministry of Health (Additional file 2), observed high malaria transmission in Gambela, Benishangul-Gumuz and Amhara Regions. By antigen comparison, *P. falciparum* API showed greater association with PfMSP-1 [Rho of 0.37 and p < 0.01 (spearman correlation)] and *P. vivax* API with PvAMA-1 (Rho 0.32 and p < 0.01).

### Seroprevalence by elevation

**Fig. 4:**
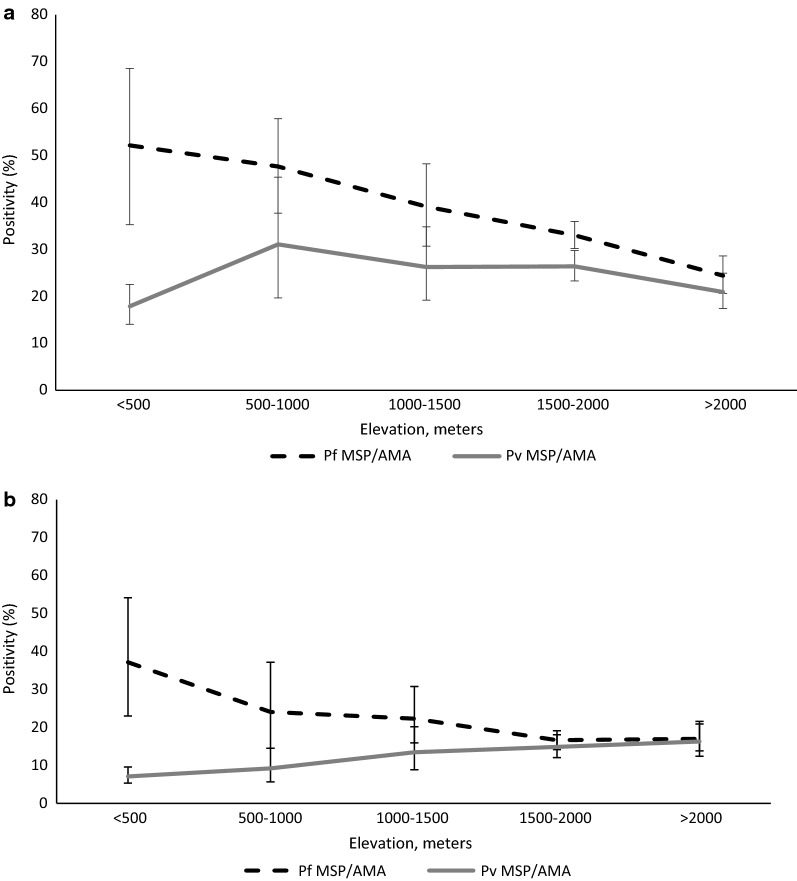
Mean seropositivity for *Plasmodium falciparum* Merozoite Surface Protien-1 (MSP-1) or Apical membrane Antigen-1 (AMA-1), and *Plasmodium vivax* MSP-1 or AMA-1 by elevation category, 2015. An individual was considered positive if they had a positive antibody response either for Merozoite Surface Protien-1 (MSP-1) or Apical Membrane Antigen-1 (AMA-1) antigens for each species. Broken line depicts *P. falciparum* (MSP-1/AMA-1) and solid line represent *P. vivax* (MSP-1/AMA-1). **a** All ages; **b** children under 5 years of age. Error bars indicate the 95% confidence interval

Seropositivity was inversely related to elevation for *P. falciparum*, with lower elevation (< 2000 m) areas having significantly higher seroprevalence compared to areas of higher elevation (2000–2500 m) [aOR 4.36 (95% CI 2.7–7.0), p < 0.01]. The highest seropositivity was observed for *P. falciparum* at elevations below 1500 m and a peak was observed at an elevation below 1000 m (Fig. 4a). A weaker inverse effect of elevation was observed for *P. vivax*. There was no significant difference in the transmission of *P. vivax* between above and below 2000 m (OR 1.5 (95% CI 0.9–2.4), p > 0.05). Among children under 5 years of age, *P. falciparum* and *P. vivax* both showed a significant although varying relationship with elevation. *Plasmodium vivax* showed direct relation with increasing elevation. High *P. vivax* seropositivity was observed at higher elevations above 1500 m (Fig. 4b). No significant effect of elevation on seropositivity was detected for the rare species, *P. malariae* and *P. ovale* (p > 0.05).

**Fig. 5:**
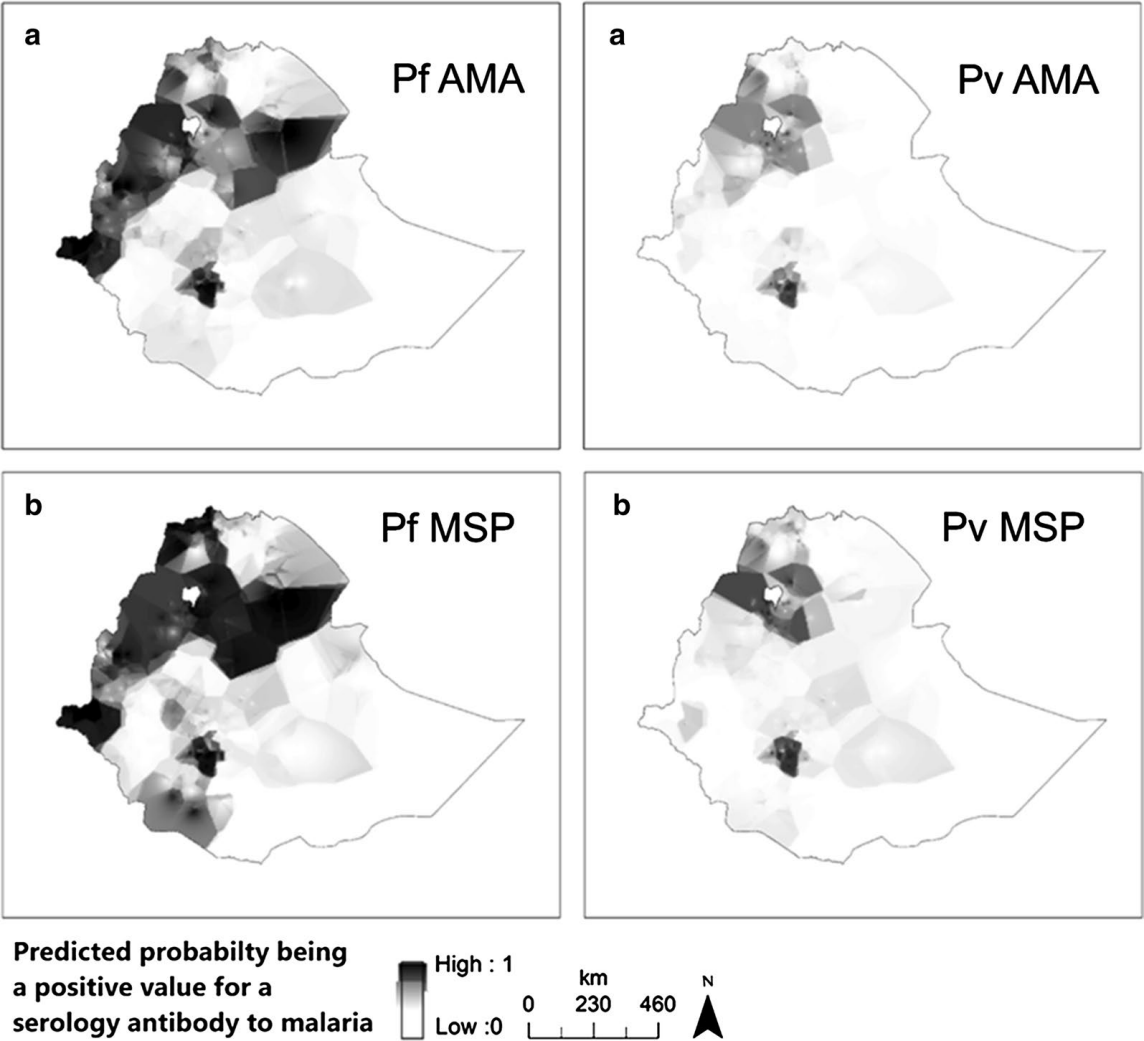
An interpolated surface map for *Plasmodium falciparum* antigen responses [**a** Apical Membrane Antigen-1 (AMA-1) and **b** Merozoite Surface Protein-1 (MSP-1)] and *Plasmodium vivax* antigen responses (**a** AMA-1 and **b** MSP-1). Darker colour shows high probability of being above the cut-off for seropositivity and lighter colour lower. Empirical bayesian kriging was used to produce the maps from serology data from samples collected during Ethiopian Malaria Indicator Survey (EMIS 2015)

Generally, seropositivity was found to vary in space, with elevation and among administrative regions, as depicted in Fig. 5. An interpolated surface map predicted differing transmission patterns for *P. falciparum* and *P.* vivax. Higher seropositivity is predicted in the northwest compared to the southeast.

### Seroconversion curves

**Fig. 6:**
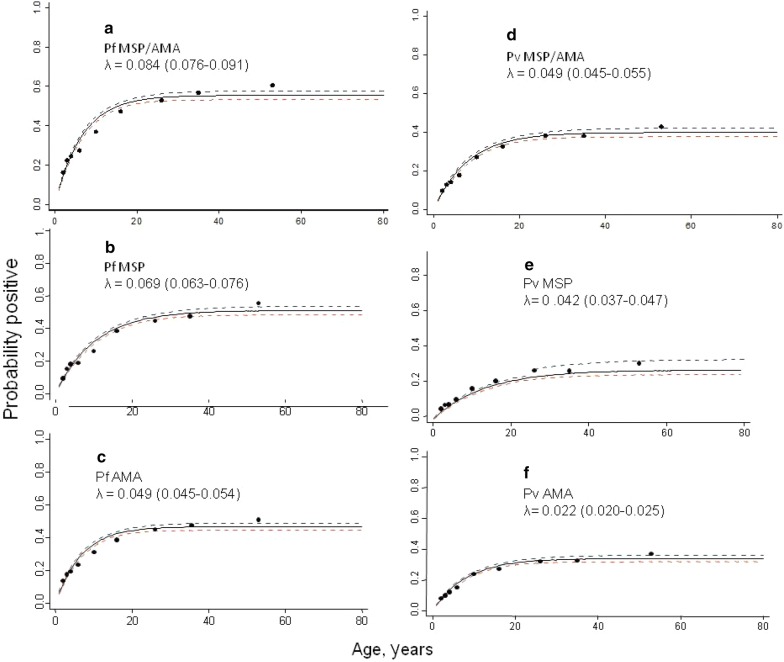
National age-seroconversion plots for antibody responses for *Plasmodium falciparum* and *Plasmodium vivax* antigens [Merozoite Surface Protien-1 (MSP-1) and Apical Membrane Antigen-1 (AMA-1)]. Y-axis represents probability of being seropositive and the X-axis age. Seroconversion curves represent the rate at which a population becomes seropositive to specific antigens resulting in seroconversion rates (SCR) or lambda (λ). In each graph points represent age seroprevalence (by deciles), unbroken line represents maximum likelihood curves and broken lines represent the 95% confidence interval. **a** and **d** depict the seroconversion rates for combined (MSP-1/AMA-1) *P. falciparum* antigen response (**a**) and for combined (MSP-1/AMA-1) *P. vivax* antigen response (**d**). Plots b and c show seroconversion rates for individual *P. falciparum* antigens MSP-1 (**b**) and AMA-1 (**c**). Plots **e** and **f** show seroconversion rates for individual *P. vivax* antigens MSP-1 (**e**) and AMA-1 (**f**)

The overall age seroconversion plot for antibody responses for both *P. falciparum* and *P. vivax* are shown in Fig. 6. In general, higher seroconversion rates were observed for *P. falciparum* compared to *P. vivax* both for the combined MSP-1/AMA-1 outcome and individual MSP-1 and AMA-1 antigens; and MSP-1 antigen show higher seroconversion rates compared to AMA-1 antigens for both species. Heterogeneity was observed across the different antigen responses. A similar trend was observed when the model was fitted to data with elevation below 2000 m and combined MSP-1 and AMA-1 antigens. Regionally, the seroconversion model was fitted to the four major administrative regions (Amhara, Oromia, SNNP, Tigray) both for *P. falciparum* and *P. vivax* MSP-1 and AMA-1 antigen responses. Heterogeneous seroconversion rates were observed over the regions (Additional files 3 and 4). For seroconversion plots, where visual inspection indicated better fit between the observed and predicted pattern of transmission, two forces of infection were compared with a single force of infection. This showed a non-significant change over around two decades for both *P. falciparum* and *P. vivax* antigen responses (p > 0.05).

## Discussion

The current study documented malaria seroprevalence in Ethiopia using samples collected from a national EMIS conducted in 2015. Of all samples analysed, 32.1% had IgG antibodies against combined *P. falciparum* MSP-1 or AMA-1 antigens, and 25.0% for *P. vivax* MSP-1 or AMA-1. The relative numbers were lower for children under 5 years of age at 18.0% for *P. falciparum* and 14.8% for *P. vivax*, suggesting a lower recent transmission in Ethiopia. Seropositivity in children under 5 years of age may accurately represent recent infection as they have had limited exposure and time to generate the cumulative antibody response seen in adults [24–26]. The serological results were cumulative antibody responses for the relatively long-term markers of transmission, MSP-1 and AMA-1 antigens [16, 22, 27–29]. The RDT results for the study samples showed 0.8% positivity for *Plasmodium*, of which 0.5% was due to *P. falciparum*.

Generally, significantly higher levels of *P. falciparum* transmission were observed in areas of lower elevation (< 2000 m). This is consistent with previous studies [1, 17, 19], and attributed to the ambient temperature and moisture requirements of the *Anopheles* vector mosquitoes [30]. This reaffirms the national malaria control programme’s strategy in which high elevation areas (> 2000 m) are considered to be areas where malaria is not transmitted and thus not targeted for vector control but only case management interventions. However, the evidence of increasing *P. vivax* seropositivity with elevation over the range of the study setting could expand the understanding of malaria transmission in Ethiopia. The relapse of *P. vivax* infections may have contributed to the high *P. vivax* prevalence observed at higher elevations [31–35]. Travel history is reported to be a risk factor for malaria exposure in Ethiopia [24–26], but in the EMIS-2015 only 2% reported travel history a month prior to the survey [12].

Seropositivity was high for children under 5 years of age, particularly in the Gambela and Benishangul Gumuz Regions, although was lower than in adults. These show higher malaria transmission that may need a more intensive approach for malaria control in the affected areas. A larger proportion of males than females were seropositive for both *P. falciparum* and *P. vivax*. Increased malaria risk in men in Ethiopia has been attributed to a greater extent of outdoor, occupational exposure [24–27, 36].

A total of 8.6% of individuals were seropositive for *P. malariae* and 3.1% for *P. ovale*. Variable trends of proportion seropositive were observed in the regions for both species. These findings, albeit with high proportion, support previous reports of sporadic occurrence of *P. malariae* and *P. ovale* that may be responsible for up to 1% of the malaria incident cases in Ethiopia [30, 37–40]. Currently there is no reporting system for these *Plasmodium* species, *P. malariae* and *P. ovale*, in Ethiopia. Antibody cross-binding among the different MSP-1 antigens has been documented as non-substantial [18, 41]. In the present study, data was analysed using an additional conservative approach to defining seropositivity to rule out the possibility of IgG cross-binding, thus reporting the most conservative estimates for comparison. Using this approach would potentially increase specificity but decrease sensitivity. The overall seropositivity, although reduced, was 5.1% for *P. malariae* and 1.5% for *P. ovale*, with the regional trend and pattern of transmission remaining similar.

The use of serological methods in measuring malaria transmission in low-transmission settings has been documented in a number of studies [15, 16, 27, 36, 42, 43]. Application of serology methods was suggested for Ethiopia because of the very low prevalence of malaria through RDT and microscopy methods across several national surveys [27, 36, 42]. The estimate was too low to track progress over the years or give detailed information about differences in malaria burden by region or risks of infection due to demographic or behavioural factors. By describing exposure to malaria over a longer period, serology data were able to better document differences in exposure between age groups, regions and by elevation.

The findings show the magnitude of transmission intensity was highly variable among different regions and elevations in Ethiopia. Yalew et al. reported similar trends with 30.0 and 21.8% seroprevalence for *P. falciparum* and *P. vivax*, respectively, for the Amhara region in 2013 [36]. The current study reported 32.1% for *P. falciparum* and 25.0% for *P. vivax* for the same region. The slight variation in seropositivity may be attributed to the different type of laboratory assay used. The challenge of comparing data from ELISA and bead-based multiplex serology has been investigated previously [15]. Results from multiplex serology were more robust, sensitive and ideal for large-scale surveys compared to ELISA [15]. Heterogeneity of response for the different antigens was demonstrated in several studies. Ashton et al. [27] described MSP-1 antigens to be better biomarkers for *P. falciparum,* although Cook et al. [16] reported AMA-1 to be better for measuring *P. falciparum* in low-transmission settings. Characterization of antigen markers may be required to further qualify the serology methods [44]. The current finding was in agreement with the API data from 2014 obtained through routine surveillance. Although routine surveillance can provide crucially important data for decision making for countries at lower transmission, additional biomarkers to track malaria burden can serve as ancillary data as surveillance systems continue to improve and dynamically evolve in completeness and quality.

The study had several limitations. Only 65% of DBS samples from the EMIS-2015 survey were available for serological analysis. Although no systematic bias was introduced and frequencies were weighted, the representativeness of this sample at the national and regional levels should be interpreted cautiously. The data were insufficient to produce refined spatial heat maps and the maps produced show only general trends. Samples collected are assumed to reflect the malaria exposure of their respective locations and did not account for potential population movement. Although the survey reported very low recent travel history, past travel encompassing the previous harvest season as well as previous years were not captured and could reflect past malaria exposure risk from a different locale. This study assessed long-term markers of transmission, but further characterizing antigens based on local eco-epidemiology is recommended to identify and select specific short- and long-term markers.

## Conclusion

The current study explored the malaria burden and transmission intensity using serological markers of exposure to four malaria species. It documented the magnitude, spatial distribution and dynamics of transmission over differing elevation, administrative regions, and age groups using samples collected during a national programme evaluation survey with minimal sample preparation time and cost. These findings confirmed the National Malaria Control Programme data of the relative high transmission in the Gambela and Benishangul-Gumuz Regions and highlighted gaps in the current understanding of malaria epidemiology. The increase of *P. vivax* seropositivity with elevation and the presence of *P. malariae* and *P. ovale* in all administrative regions (given *P. ovale* requires hypnozoiticidal treatment) may require programmatic consideration in selecting diagnostic tools and appropriate treatment guidelines. These results can be used as baseline data to evaluate the recent malaria elimination efforts in Ethiopia especially as routine surveillance systems are progressively being strengthened.

## Additional files

**Additional file 1.** Percent malaria seropositivity and Odds Ratio (OR) by sex, age and elevation for each species in malaria endemic areas of Ethiopia, 2014.

**Additional file 2.** An Interpolated surface map for *P. falciparum* (left) and *P. vivax* (right). Empirical bayesian kriging was used to produce the maps from the Annual Parasite Incidence data in Ethiopia (API 2014).

**Additional file 3.** Regional age-seroconversion plots for antibody responses for *Plasmodium falciparum* antigens. Y-axis represents probability of being seropositive and the X-axis age. Seroconversion curves represent the rate at which a population become seropositive to specific antigens resulting in seroconversion rates (SCR) or lambda (λ). In each graph points represent age seroprevalence (by deciles), unbroken line represents maximum likelihood curves and broken lines represent the 95% confidence interval. Plots A and B depict the seroconversion curves for *P. falciparum* antigens response to MSP-1 (A) and AMA-1(B) for the major four regions Tigray, Amhara, Oromia and Southern Nations and Nationalities People’s Regions.

**Additional file 4.** Regional age-seroconversion plots for antibody responses for *P. falciparum* antigens. Y-axis represents probability of being seropositive and the X-axis age. Seroconversion curves represent the rate at which a population become seropositive to specific antigens resulting in seroconversion rates (SCR) or lambda (λ). In each graph points represent age seroprevalence (by deciles), unbroken line represents maximum likelihood curves and broken lines represent the 95% confidence interval. Plots A and B depict the seroconversion curves for *P. vivax* antigens response to MSP-1 (A) and AMA-1 (B) for the major four regions, Tigray, Amhara, Oromia and Southern Nations and Nationalities People’s Region (SNNPR).

## Data Availability

The datasets used and/or analysed during the current study are available from the corresponding author on reasonable request.

## Abbreviations

AMA-1: Apical Membrane Antigen-1
API: annual parasite incidence
aOR: adjusted odds ratio
BG: Benishangul-Gumuz
BSA: bovine serum albumin
CDC: Center for Disease Control
CI: confidence interval
DBS: dried blood spot
EA: enumeration area
ELISA: Enzyme Linked Immunosorbent Assay
EMIS: Ethiopia Malaria Indicator Survey
FMM: Finite Mixture Model
GST: Gluthathione-S-Transferase
HRP2: Histidin Rich Protein 2
IgG: Immune globulin G
LSHTM: London School of Hygiene and Tropical Medicine
MFI: mean fluorescence intensity
MSP-1: Merozoite Surface Protein-1
RERRC: National Ethics and Research Review Committee
OR: odds ratio
PBS: phosphate buffered saline
PCR: polymerase chain reaction
pLDH: plasmodium lactate dehydrogenase
RDT: rapid diagnostic test
RFLP: restriction fragment length polymorphism
RT: room temperature
SCR: seroconversion rate
SNNPR: Southern Nations and Nationalities People’s Region
WRAIR: Walter Reed Army Institute of Research
