# Supplementary material for: Multiplex serology demonstrate cumulative prevalence and spatial distribution of malaria in Ethiopia
Source: Malar J. 2019 Jul 22;18:246. doi: 10.1186/s12936-019-2874-z (PMC6647069; doi:10.1186/s12936-019-2874-z)
Supplement: Supplementary file 1 — Additional file 1. Percent malaria seropositivity and Odds Ratio (OR) by sex, age and elevation for each species in malaria endemic areas of Ethiopia, 2014. [file 12936_2019_2874_MOESM1_ESM.doc]

**Additional files**

Additional file 1. Percent malaria seropositivity and Odds Ratio (OR) by sex, age and elevation for each species in malaria endemic areas of Ethiopia.

|  | *P. falciparum* | | | *P. vivax* | | |
| --- | --- | --- | --- | --- | --- | --- |
|  |  |  |  |  |  |  |
|  | % Seropositive (MSP-1 or AMA-1) | Unadjusted OR (95% CI) | Adjusted OR (95% CI)‡ | % Seropositive (MSP-1 or AMA-1) | Unadjusted OR (95% CI) | Adjusted OR (95% CI)‡ |
| Sex |  |  |  |  |  |  |
| Female | 30.9 (28.3-33.6) | Ref | Ref | 23.6 (21.1-26.3) | Ref | Ref |
| Male | 33.3 (30.4-36.2) | 1.1 (1.0-1.2) | 1.3 (1.1-1.4)** | 26.4 (23.4-29.7) | 1.2 (1.0-1.4) | 1.3 (1.1-1.5) |
| Age (Years) |  |  |  |  |  |  |
| <5 | 18.0 (16.0-20.2) | Ref | Ref | 14.8 (12.7-17.3) | Ref | Ref |
| 5-15 | 31.5 (27.6-35.6) | 2.2 (1.9-2.5)* | 2.3 (2.0-2.6)* | 25.7 (22.5-29.2) | 2.5 (2.2-2.9)* | 2.5 (2.2-3.0)* |
| 15-25 | 43.7 (38.6-48.8) | 3.8 (3.3-4.5) * | 4.0 (3.5-4.7)* | 34.0 (28.7-39.8) | 4.6 (4.0-5.5)* | 4.5 (3.8-5.4)* |
| 25-50 | 50.5 (45.4-55.5) | 5.2 (4.6-5.9)* | 6.5 (5.6-7.5)* | 36.7 (32.7-40.8) | 6.6 (5.7-7.6)* | 4.9 (4.2-5.7)* |
| ≥50 | 58.2 (52.0-64.2) | 6.3 (5.1-7.8)* | 8.0 (6.4-9.9)* | 43.7 (36.8-50.8) | 8.3 (6.7-10.3)* | 6.2 (5.0-7.6)* |
| Elevation (meters) |  |  |  |  |  |  |
| >2000 | 24.4 (20.6-28.6) | Ref | Ref | 20.9 (17.4-24.9) | Ref | Ref |
| 1000-2000 | 34.3 (31.437.3) | 1.6 (1.3-2.1)* | 1.8 (1.4-2.4)* | 26.4 (23.4-29.5) | 1.4 (1.0-1.8) | 1.5 (1.1-2.0)** |
| <1000 | 49.9 (40.3-59.5) | 3.1 (2.0-4.8)* | 4.4 (2.7-7.0)* | 24.6 (17.2-33.9) | 1.2 (0.7-2.1) | 1.5 (0.9-2.3) |

**P-values <0.05; * P-values <0.01 ‡ Controlled for listed risk factors
