# Supplementary material for: Multiplex serology demonstrate cumulative prevalence and spatial distribution of malaria in Ethiopia
Source: Malar J. 2019 Jul 22;18:246. doi: 10.1186/s12936-019-2874-z (PMC6647069; doi:10.1186/s12936-019-2874-z)
Supplement: Supplementary file 2 — Additional file 2. An Interpolated surface map for P. falciparum (left) and P. vivax (right). Empirical bayesian kriging was used to produce the maps from the Annual Parasite Incidence data in Ethiopia (API 2014). [file 12936_2019_2874_MOESM2_ESM.doc]

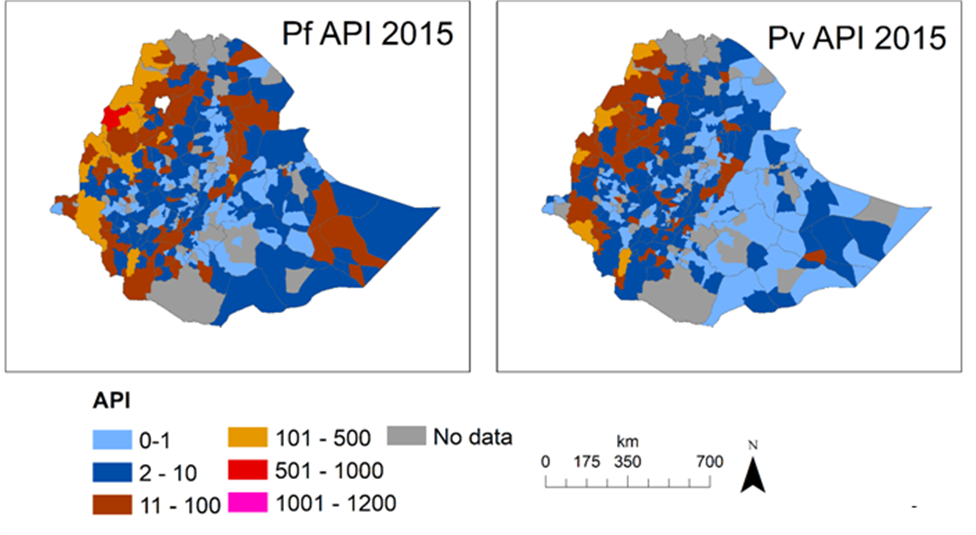


Additional file 2. An Interpolated surface map for *P. falciparum* (left) and *P. vivax* (right). Empirical bayesian kriging was used to produce the maps from the Annual Parasite Incidence data in Ethiopia (API 2015)
