# Supplementary material for: Multiplex serology demonstrate cumulative prevalence and spatial distribution of malaria in Ethiopia
Source: Malar J. 2019 Jul 22;18:246. doi: 10.1186/s12936-019-2874-z (PMC6647069; doi:10.1186/s12936-019-2874-z)
Supplement: Supplementary file 4 — Additional file 4. Regional age-seroconversion plots for antibody responses for P. falciparum antigens. Y-axis represents probability of being seropositive and the X-axis age. Seroconversion curves represent the rate at which a population become seropositive to specific antigens resulting in seroconversion rates (SCR) or lambda (λ). In each graph points represent age seroprevalence (by deciles), unbroken line represents maximum likelihood curves and broken lines represent the 95% confidence interval. Plots A and B depict the seroconversion curves for P. vivax antigens response to MSP-1 (A) and AMA-1 (B) for the major four regions, Tigray, Amhara, Oromia and Southern Nations and Nationalities People’s Region (SNNPR). [file 12936_2019_2874_MOESM4_ESM.doc]

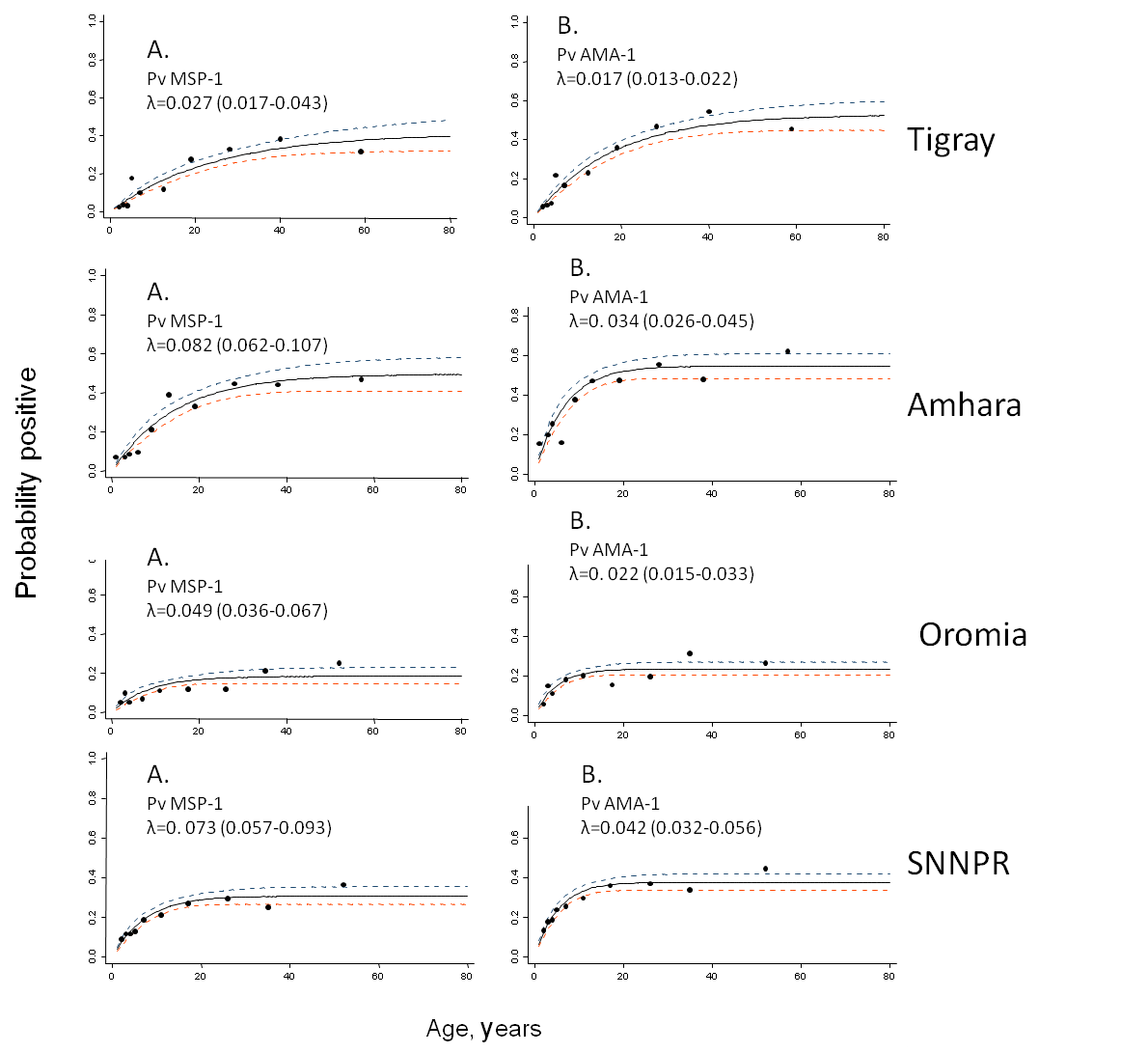


Additional file 4: Regional age-seroconversion plots for antibody responses for *P. falciparum* antigens. Y-axis represents probability of being seropositive and the X-axis age. Seroconversion curves represent the rate at which a population become seropositive to specific antigens resulting in seroconversion rates (SCR) or lambda (λ). In each graph points represent age seroprevalence (by deciles), unbroken line represents maximum likelihood curves and broken lines represent the 95% confidence interval. Plots A and B depict the seroconversion curves for *P. vivax* antigens response to MSP-1 (A) and AMA-1 (B) for the major four regions, Tigray, Amhara, Oromia and Southern Nations and Nationalities People’s Region (SNNPR).
